# Supplementary material for: Priorities for developing stroke care in Ireland from the perspectives of stroke survivors, family carers and professionals involved in stroke care: A mixed methods study
Source: PLoS One. 2024 Jan 19;19(1):e0297072. doi: 10.1371/journal.pone.0297072 (PMC10798447; doi:10.1371/journal.pone.0297072)
Supplement: S1 File — (DOCX) [file pone.0297072.s001.docx]

S1 File Consolidated criteria for reporting qualitative studies (COREQ): 32-item checklist

| Item | Guide questions/description | Section |
| --- | --- | --- |
| **Domain 1: Research team and reflexivity** |  |  |
| *Personal Characteristics* |  |  |
| 1. Interviewer/facilitator | Which author/s conducted the interviews? | Data collection and analysis |
| 2. Credentials | What were the researcher’s credentials? | Data collection and analysis |
| 3. Occupation | What was their occupation at the time of the study? | Data collection and analysis |
| 4. Gender | Was the researcher male or female? | Data collection and analysis |
| 5. Experience and training | What experience or training did the researcher have? | Data collection and analysis |
| *Relationship with participants* |  |  |
| 6. Relationship established | Was a relationship established prior to study commencement? | Participants |
| 7.Participant knowledge  of the interviewer | What did the participants know about the researcher? | See PIL in OSF deposit |
| 8. Interviewer characteristics | What characteristics were reported about the interviewer? (e.g. bias) | N/A |
| **Domain 2: study design** |  |  |
| *Theoretical framework* |  |  |
| 9. Methodological orientation | What methodological orientation was stated to underpin the study? | Data collection and analysis |
| *Participant selection* |  |  |
| 10. Sampling | How were participants selected? | Participants |
| 11. Method of approach | How were participants approached? | Participants |
| 12. Sample size | How many participants were in the study? | Results |
| 13. Non-participation | How many people refused to participate or dropped out? Why? | Results, S5 Table |
| *Setting* |  |  |
| 14. Setting of data collection | Where was the data collected? | Participants; Data Collection and Analysis |
| 15. Presence of non-participants | Was anyone else present besides the participants and researchers? | Results |
| 16. Description of sample | What are the important characteristics of the sample? | Results, S1-S5 Tables |
| *Data collection* |  |  |
| 17. Interview guide | Were questions, prompts, guides provided by the authors? | Topic guide available in OSF deposit |
| 18. Repeat interviews | Were repeat interviews carried out? If yes, how many? | Results |
| 19. Audio/visual recording | Did the research use audio or visual recording to collect the data? | Data collection and analysis |
| 20. Field notes | Were field notes made during and/or after the interview? | Data collection and analysis |
| 21. Duration | What was the duration of the interviews or focus group? | Results |
| 22. Data saturation | Was data saturation discussed? | Participants |
| 23. Transcripts returned | Were transcripts returned to participants for comment/correction? | Data collection and analysis |
| **Domain 3: analysis and findings** |  |  |
| *Data analysis* |  |  |
| 24. Number of data coders | How many data coders coded the data? | Data collection and analysis |
| 25. Description of the coding tree | Did authors provide a description of the coding tree? | Available in OSF deposit |
| 26. Derivation of themes | Were themes identified in advance or derived from the data? | Data collection and analysis |
| 27. Software | What software, if applicable, was used to manage the data? | Data collection and analysis |
| 28. Participant checking | Did participants provide feedback on the findings? | Data collection and analysis |
| *Reporting* |  |  |
| 29. Quotations presented | Were participant quotations presented to illustrate the themes? | Results, Table 1 |
| 30. Data and findings consistent | Was there consistency between the data presented and the findings? | Results, Table 1 |
| 31. Clarity of major themes | Were major themes clearly presented in the findings? | Results, Table 1 |
| 32. Clarity of minor themes | Is there a description of diverse cases or discussion of minor themes? | Results, Table 1, further information in OSF deposit |

PIL=Participant Information Leaflet, SM=Supplementary Material, T1 = Table 1

Retrieved from: Tong A, Sainsbury P, Craig J. Consolidated criteria for reporting qualitative research (COREQ): a 32-item checklist for interviews and focus groups. *International Journal for Quality in Health Care*. 2007. Volume 19, Number 6: pp. 349 – 357
