# Supplementary material for: Priorities for developing stroke care in Ireland from the perspectives of stroke survivors, family carers and professionals involved in stroke care: A mixed methods study
Source: PLoS One. 2024 Jan 19;19(1):e0297072. doi: 10.1371/journal.pone.0297072 (PMC10798447; doi:10.1371/journal.pone.0297072)
Supplement: S1 Table — (DOCX) [file pone.0297072.s002.docx]

S1 Table. Survivor Interviewee Profile (n=18)

| ID | Age Group | Sex | Area | Interview Type | Stroke Type | Communication or cognitive problems | Mobility | Support needed with self-care |
| --- | --- | --- | --- | --- | --- | --- | --- | --- |
| C_01* | 60-64 | M | West Dublin | Phone | I | Aphasia | Limited - uses wheelchair sometimes | Needs some support |
| S_01 | 65-69 | F | West Dublin | Phone | H | Mild cognitive issues | Good | Independent |
| S_02 | 60-64 | M | North West | Phone | O | Mild cognitive and communication issues | Good | Independent |
| S_03 | 65-69 | F | South East | MS Teams | I | Mild cognitive issues | Limited due to life-long disability | Independent |
| S_04 | <50 | F | West | MS Teams | I | Cognitive issues | Limited | Needs some support |
| S_06 | 50-59 | M | West | Phone | I | None mentioned | Good | Independent |
| S_07 | 50-59 | M | North East | Phone | H | None mentioned | Good | Independent |
| S_09 | 80-89 | F | North West | Phone | H | Mild cognitive issues | Some issues | Independent |
| S_10 | 65-69 | F | West | Phone | I | None mentioned | Some issues | Independent |
| S_11 | 50-59 | M | West | Phone | I | None mentioned | Good | Independent |
| S_12 | 75-79 | F | Midlands | Phone | O | Aphasia, hearing problems, mild cognitive issues | Some issues | Independent |
| S_13 | 75-79 | F | North Dublin | Phone | I | None reported | Some issues | Independent |
| S_14 | 80-89 | F | South West | Phone | H | Mild aphasia and cognitive issues | Some issues | Needs some support |
| S_15 | <50 | M | North East | MS Teams | I | Aphasia | Good | Independent |
| S_16 | 50-59 | M | South East | MS Teams | O | Aphasia | Good | Independent |
| S_17 | 70-74 | M | South West | Phone | H | None mentioned | Limited - uses a wheelchair | Needs high level, 24hr support |
| S_18 | 75-79 | M | South West | Phone | H | Hearing impairment | Limited | Needs some support, e.g., with showering |
| S_19 | 70-74 | M | West | Phone | H | Mild cognitive issues | Limited - uses a wheelchair | Needs high level support -lives in a nursing home |

*As this was a survivor and carer dyad, the carer was the main interviewee and the interview was coded under a Carer id

I=Ischaemic, H=Haemorrhagic, O = Other
