# Supplementary material for: Priorities for developing stroke care in Ireland from the perspectives of stroke survivors, family carers and professionals involved in stroke care: A mixed methods study
Source: PLoS One. 2024 Jan 19;19(1):e0297072. doi: 10.1371/journal.pone.0297072 (PMC10798447; doi:10.1371/journal.pone.0297072)
Supplement: S2 Table — (DOCX) [file pone.0297072.s003.docx]

S2 Table. Family/main carer Interviewee Profile (n=13)

| ID | Age Group | Gender | Area | Interview Type | Stroke Type | Relationship | Survivor mobility | Support needed with self-care |
| --- | --- | --- | --- | --- | --- | --- | --- | --- |
| C_01 | 60-64 | F | West Dublin | Phone | I | Wife | Limited - uses wheelchair sometimes | Needs some support |
| C_02 | 70-74 | F | South Dublin | Phone | H | Wife | Very limited mobility, wheelchair user | Needs high level of support |
| C_03 | 50-59 | F | South Dublin | MS Teams | I | Daughter | Very limited mobility, wheelchair user | Needs high level of support |
| C_04 | <50 | F | North West | MS Teams | I | Daughter | Very limited mobility, mostly bed-bound | Needs high level of support |
| C_05 | 75-79 | F | North East | Phone | H | Wife | Some mobility issues | Needs some support |
| C_06 | 65-69 | F | West | Phone | O | Partner | Some mobility issues | Needs some support |
| C_08 | 70-74 | F | West | Phone | O | Wife | Very limited mobility, has dementia | Needs high level of support |
| C_09 | 60-64 | F | South Dublin | Phone | O | Wife | Limited mobility | Needs high level of support |
| C_10 | 50-59 | F | South Dublin | MS Teams | I | Sister | Some mobility issues | Independent |
| C_11 | 60-64 | F | South West | MS Teams | I | Wife | Very limited mobility | Needs high level of support |
| C_12 | 60-64 | F | South West | Phone | H | Wife | Very limited mobility, wheelchair user | Needs high level of support |
| C_14 | <50 | F | Midlands | Phone | O | Daughter | Good mobility | Needs high level of support – lives in a nursing home, has dementia |
| S_12* | NA | M | Midlands | Phone | O | Husband | Some mobility issues | Independent |

*As this was a survivor and carer dyad, the survivor was the main interviewee and the interview was coded under a Survivor id

I=Ischaemic, H=Haemorrhagic, O = Other
