# Supplementary material for: Priorities for developing stroke care in Ireland from the perspectives of stroke survivors, family carers and professionals involved in stroke care: A mixed methods study
Source: PLoS One. 2024 Jan 19;19(1):e0297072. doi: 10.1371/journal.pone.0297072 (PMC10798447; doi:10.1371/journal.pone.0297072)
Supplement: S3 Table — (DOCX) [file pone.0297072.s004.docx]

S3 Table. Senior leader interviewee profile (n=8)

| ID | Position | Gender | Interview type |
| --- | --- | --- | --- |
| SL_01 | Consultant and national clinical lead | W | Phone |
| SL_02 | Consultant and national clinical lead | M | Face-to-face |
| SL_03 | Consultant and national clinical lead | W | MS Teams |
| SL_04 | Senior staff member in voluntary organisation | M | MS Teams |
| SL_05 | Senior psychologist working in stroke | W | Phone |
| SL_06 | Programme manager and stroke clinical nurse specialist | W | MS Teams |
| SL_07 | Senior academic researcher and physiotherapist | W | MS Teams |
| SL_08 | Consultant and national clinical lead | W | MS Teams |
