# Supplementary material for: Priorities for developing stroke care in Ireland from the perspectives of stroke survivors, family carers and professionals involved in stroke care: A mixed methods study
Source: PLoS One. 2024 Jan 19;19(1):e0297072. doi: 10.1371/journal.pone.0297072 (PMC10798447; doi:10.1371/journal.pone.0297072)
Supplement: S4 Table — (DOCX) [file pone.0297072.s005.docx]

S4 Table. Phase 1 Survey Participants Profile (Professionals) (n=80)

| **Type of Professional** | **N** | **%** |
| --- | --- | --- |
| Allied Health Professional | 49 | 61 |
| Health Professional (e.g. doctor, nurse, pharmacist) | 17 | 21 |
| Other (e.g., manager, policy maker, researcher, working in a charity) | 14 | 18 |
| **Geographical Area** | **N** | **%** |
| Dublin | 41 | 53 |
| West | 25 | 32 |
| East or Midlands | 8 | 10 |
| Multiple areas | 4 | 5 |
| **Service area** | **N** | **%** |
| Acute services | 45 | 56 |
| Rehabilitation services | 24 | 30 |
| Inpatient services | 17 | 21 |
| Outpatient services | 10 | 13 |
| Early Supported Discharge | 8 | 10 |
| Community | 8 | 10 |
| Not involved in care delivery | 7 | 9 |
| Other, inc. helplines and other voluntary organisation activity, social care | 5 | 6 |

Notes: Percentages may not add to 100 as a result of rounding. There were also three stroke survivors and five main carers who took part in the Phase 2 survey, but their demographic details are not reported to preserve anonymity.
