# Supplementary material for: Priorities for developing stroke care in Ireland from the perspectives of stroke survivors, family carers and professionals involved in stroke care: A mixed methods study
Source: PLoS One. 2024 Jan 19;19(1):e0297072. doi: 10.1371/journal.pone.0297072 (PMC10798447; doi:10.1371/journal.pone.0297072)
Supplement: S5 Table — (DOCX) [file pone.0297072.s006.docx]

S5 Table. Phase 2 Survey Participants Profile

| **Geographical Area** | **Professionals** | | **Survivors** | | **Carers** | |
| --- | --- | --- | --- | --- | --- | --- |
|  | **N** | **%** | **N** | **%** | **N** | **%** |
| Dublin | 18 | 43 | 6 | 18 | 8 | 47 |
| East or Midlands | 10 | 24 | 11 | 33 | 5 | 24 |
| West | 15 | 36 | 16 | 43 | 4 | 29 |
| Total | 42 **^a^** | 100 | 33 | 100 | 17 | 100 |
| **Type of Professional** | **N** | **%** |  |  |  |  |
| Health and social care professional e.g. speech and language therapist, physiotherapist, social worker) | 21 | 50 |  |  |  |  |
| Researcher, working in charity/advocacy, manager or other | 13 | 31 |  |  |  |  |
| Medical professional (e.g. GP, consultant) or Nurse | 8 | 19 |  |  |  |  |
| Total | 42 | 100 |  |  |  |  |
| **Service Area** |  |  |  |  |  |  |
| Acute Services | 22 | 52 |  |  |  |  |
| Community, Outpatient, Social Care, ESD | 15 | 36 |  |  |  |  |
| Inpatient services | 10 | 24 |  |  |  |  |
| Not involved in care delivery or Other | 8 | 19 |  |  |  |  |
| Rehabilitation services | 15 | 36 |  |  |  |  |
|  | 42 **^a^** | **100** |  |  |  |  |
| **Age Group** |  |  | **N** | **%** | **N** | **%** |
| Under 50 years |  |  | 13 | 39 | 5 | 29 |
| 50-59 years |  |  | 9 | 27 | 6 | 35 |
| 60-69 years |  |  | 6 | 18 | 5 | 29 |
| 70+ years |  |  | 5 | 15 | 1 | 6 |
| **Total** |  |  | **33** | **100** | **17** | **100** |
| **Sex** |  |  |  |  |  |  |
| Man |  |  | 16 | 48 | 2 | 12 |
| Woman |  |  | 17 | 52 | 15 | 88 |
| **Total** |  |  | **33** | **100** | **17** | **100** |
| **Year of Stroke / Starting to Care** |  |  |  |  |  |  |
| 2010-2019 |  |  | 20 | 61 | 6 | 35 |
| 2020-2022 |  |  | 12 | 36 | 8 | 47 |
| Before 2010 |  |  | 1 | 3 | 2 | 18 |
| **Total** |  |  | **33** | **100** | **17** | **100** |

**^a^**Professional participants could choose more than one area

ESD = Early Supported Discharge

Note: 3 survivors and 2 carers did not provide demographic information

Non-response: Precise response rates were not possible to calculate due to the anonymous nature of the survey. Five participants requested postal surveys, and three requested interviews, and all were completed and included. For the online survey, 134 email and 12 text message invitations were sent. The consent screen of the survey was completed by 116 people, and 87 of those went on to complete the survey. Of the 29 who did not complete, 16 were professionals, 7 were survivors, 4 were family carers, and 2 did not indicate their participant type.
